# COVID-19 Vaccine Literacy of Family Carers for Their Older Parents in Japan

**DOI:** 10.3390/healthcare9081038

**Published:** 2021-08-12

**Authors:** Hiroko Costantini

**Affiliations:** Observatoire Sociologique du Changement (OSC), Sciences Po and Oxford Institute of Population Ageing, University of Oxford, Oxford OX1 4BH, UK; hiroko.umegakicostantini@sciencespo.fr

**Keywords:** vaccine literacy, Japan, COVID-19, family carers for older adults, sustainable ageing society, health communications, mass media

## Abstract

In super-ageing Japan, COVID-19 vaccinations were starting to reach older people as of June 2021, which raises the issue of vaccine literacy. This study focuses on family members who work and also care for their older parents, as they are at risk of COVID-19 and also risk transmitting COVID-19 to the parents they care for and potentially influencing their parents’ vaccine uptake. Such family carers are central to the approach in Japan to achieving a sustainable and resilient society in response to ageing. Contrasting family carers’ COVID-19 vaccine literacy with their overall health literacy provides insights into their preparedness for COVID-19 vaccinations. The purpose of this study is to understand how vaccine literacy, compared to health literacy, varies across family carers and the sources of information they use. Through a cross-sectional online survey, family carers’ vaccine literacy, health literacy and their sources of information, including mass media, social media, health and care professionals, family, colleagues, friends, and others, were assessed. The participants’ (*n* = 292) mean age was 53, with 44% women, and an average of 8.3 h per week caring for their parents. Notwithstanding the increased risks from COVID-19 with age, COVID-19 vaccine literacy relative to health literacy for older family carers is lower on average, higher with increased provision of care, and more variable, resulting in a substantial proportion of older family carers with relatively low vaccine literacy. At this stage of vaccine rollout in Japan, family carers’ sources of information to inform COVID-19 vaccine literacy is distinct, including more national and local mass media versus less health and care professionals and informal networks, which indicates the importance of tailored health communication strategies to enhance vaccine literacy

## 1. Introduction

Vaccinations are a central aspect of addressing the COVID-19 pandemic, with unprecedented rapid development of several vaccines and initial heterogeneous deployment across countries. The preventative collective benefit of vaccinations depends on individuals’ voluntary vaccination decisions. Indeed, in the context of the COVID-19 pandemic, understanding intentions to vaccinate [1] and thereafter vaccination rates are a key issue. This brings to the fore the notion of ‘vaccine literacy’, related to although distinct from health literacy, encompassing an understanding of the potential benefits from vaccination, the risks of side effects, as well as the economic costs and organizational process to access vaccination [2]. In turn, vaccine literacy is contingent on personal circumstances as well as the broader societal context, thus contributing to shape intentions to vaccinate and ultimately vaccine uptake [3].

In the context of COVID-19, the importance of health literacy, in particular critical literacy necessary to deal with the uncertainty and complexity of the pandemic as well as the torrent of more or less accurate information, has been discussed [4,5]. With the increased role of online sources of information, the concept and roles of e-health literacy come to the fore [6,7], which raises emphasis on the sources of information used to inform health literacy [8]. Furthermore, the link between health literacy and response to COVID-19 is recognized to vary substantially across diverse segments of the population [9,10]. There is also emphasis on how health literacy contributes to vaccination outcomes, although with diverse pathways of impact [1]. The link between health literacy and vaccinations depends on specific circumstances, including which vaccine, age, and social-cultural conditions, and information related to vaccines is deemed to be relatively complex in the context of health-related information [3].

Thus, for specific instances of vaccination there is value in understanding overall health literacy as well as vaccine literacy [11]. While the same functional, communication and critical literacy components comprise health and vaccine literacy [11,12], the importance depends on context, such as communication and critical literacy viewed as important in the novel, changing, complex COVID-19 pandemic [13,14]. Thus, particularly for COVID-19 vaccinations, there is value in considering both concepts in a given situation: health literacy as a person’s broad ability to understand health-related issues, and vaccine literacy as a person’s specific understanding at a point in time for a particular vaccination, which is particularly pertinent in considering rapidly emerging novel COVID-19 vaccinations.

Notwithstanding the recognition of the importance of health literacy and vaccine literacy, to date there has been a gap in assessing both underlying health literacy as well as vaccine literacy. Indeed, a person’s health literacy provides a relevant baseline against which to compare their vaccine literacy in a specific instance: that is, relevant gaps in vaccine literacy are not only low levels of vaccine literacy absolutely but also relative to health literacy. Hence, this research aims to shed light on the differences between vaccine and health literacy in the context of COVID-19 vaccinations. Furthermore, distinguishing overall health literacy from specific vaccine literacy enables distinguishing of information sources used to address these literacies, which is important when considering potential interventions to address health and vaccine literacy. Given the increasingly severe impact of COVID-19 with age, super-aging Japan where the population aging rate is highest in the world with 28.4% over 65 in 2020 [15] provides a relevant context to understand vaccine literacy. Notwithstanding the health risks due to COVID-19 and the knock-on effects of measures to contain COVID-19 [16], skepticism towards vaccines is relatively prevalent and the likelihood of taking up a COVID-19 vaccine, based on January 2021 survey data, is around 62% of adults [17]. Vaccine access has increased relatively slowly [18], with as of June 1 2021 vaccines only available to adults over 65 years old among whom 21.9% have received one shot and 2.0% two shots of vaccine [19]. Furthermore, for the majority of older adults requiring care the main providers of care are family members: among the 4.9 million people receiving care, 1.0 million are in care facilities and 3.9 million receive care at home [20]. Also, according to a major government survey [21], among those receiving care at home, 12.1% receive care from a service provider and 67.8% from a family member (and for 20.1% from others or the main carer is not identified). Of those receiving care from a family member, 54.4% live with their main care provider who is primarily their spouse (23.8%) or adult child (20.7%). Such family members self-identify as primary carers, as the survey does not provide a definition of primary carers, which points to the central role of family carers for older adults.

Furthermore, among adults, family carers who work as well as care for their parents are an important group to understand as they may influence vaccine decisions of their older parents whom they care for. Also, in the context of the highly transmittable COVID-19 virus, they indirectly connect their parents to other extended family members, work colleagues, friends, and neighbors. Importantly, their vaccination would be an important prevention for their parents. Considering such family carers, relative to past practices in which non-working daughters-in-law were the main carers for their parents-in-law, care is increasingly provided by male and female family members who also work, which is rapidly becoming the norm as aging of society progresses [22]. At the same time, the Japanese Community-based Integrated Care Systems for older people are under development, with the aim of creating a sustainable society in response to trends in ageing and that is inclusive of older people: these systems are designed with care by family members as the base, next with support from the community, and thereafter public care services [23,24]. Thus, family carers who also work are central to care for older relatives in the community. Such carers’ vaccine literacy depends on their sources of information [25], which importantly should be considered broadly [26] so including informal sources from friends and family, professional sources such as doctors and care professionals, social media and mass media. In turn, this places emphasis on the health communications associated with achieving health and vaccine literacy through appropriately targeted communication strategies, for which an understanding of whom to target and through which channels is key. Thus, the objective of this study is to understand, based on a cross-sectional online survey of family carers, the patterns in vaccine literacy relative to health literacy and the primary sources of information vaccine literacy is based on.

## 2. Materials and Methods

### 2.1. Participants

The cross-sectional survey was conducted through an online research service company, running from 15 May–2 June 2021. The survey reached a random sample of adults aged 20–70 years old (*n* = 5000) drawn from the online research company database that is representative of the population at large. Among these, the inclusion criteria were for survey respondents who care for older parents and also work or are taking leave from work due to provision of care: this resulted in *n* = 329 respondents, among whom a substantial majority completed the survey with usable entries, in particular on their health literacy, vaccine literacy and sources of media and information used to understand COVID-19 vaccines, yielding a sample used of *n* = 292.

### 2.2. Health Literacy and Vaccine Literacy

Health literacy was assessed based on the approach developed by Ishikawa et al. [27,28], with original scale items available in Japanese and versions of this instrument used in different health care contexts. This instrument has been validated for use in online surveys, based on a version in Italian used to assess COVID-19 vaccination [14]. The instrument is based on considering different aspects of health literacy: functional literacy, which is primarily about ability to read and understand information; communication literacy, which focuses on accessing information from diverse sources and explaining to others; and critical literacy, which is about assessing the quality and applicability of information to inform decision-making [12]. As in Japan the literacy rate is very high, functional literacy is taken as given. Hence, the nine-item scale used enables self-assessment of health communication literacy (five items) and health critical literacy (four items), each rated on a four-point score, from not at all (=1) to very well (=4), with the average score computed as a measure of health literacy. Participants were asked to answer the nine-item scale for their level of health literacy in general, not specific to the current moment.

In contrast, for vaccine literacy the questions asked were with regards to the present moment with regard to COVID-19 vaccination. To assess COVID-19 vaccine literacy, the Ishikawa et al. nine-item scale for health literacy was used as a base, with phrasing adapted to reflect the focus on the present understanding of vaccine literacy as related to COVID-19 vaccines. Thus, vaccine literacy was also rated on a four-point score, from not at all (=1) to very well (=4) for each item.

The difference in scores between vaccine literacy and health literacy was constructed: this difference for each individual can range from −3 to +3 (as each of vaccine and health literacy range from 1 to 4), with a positive (negative) number indicating higher (lower) vaccine versus health literacy. The difference between vaccine and health literacy is of interest. The health literacy measure reflects individual aspects that motivate the need for health literacy, such as health conditions, and individual propensity to access and use health-related information in general, not only due to COVID-19 or COVID-19 vaccines, as well as individual interpretations of the scales. Thus, overall health literacy provides a useful individual baseline against which to assess individual’s current vaccine literacy: in the context of a crisis, whether individuals are addressing the need for COVID-19 vaccine-related information to a sufficient degree, in particular relative to their baseline health literacy. Thus, the main variable of interest in the analysis is the difference between the scores for vaccine literacy and health literacy.

### 2.3. Media and Information Sources

To understand what informs participants’ sense of vaccine and health literacy, participants were asked to indicate the main sources of information they used spanning information from media, health and care services, and personal connections. In particular, options included national and local versions of broadcast and print media, social media, podcasts, medical and care service staff and professionals, as well as family members, friends, colleagues, neighbors, and ‘others’ for participants to define. Furthermore, participants were asked to assess the overall usefulness and reliability of their information sources on a five-point scale, from not at all (=1) to very well (=5).

### 2.4. Other Variables

The other variables describe aspects of the respondents’ individual, family and care context. These demographic and care-related variables are: age; household income; and hours of care provided to parents; gender; marital status (either married or not married); whether or not they have children; educational level; whether full-time employees or taking care leave; parental care needs, assessed based on the government scale of seven care needs categories, from needing light daily-living support to requiring full-time assistance; and distance from their parents, including whether living together, within walking distance, or a range of travel times from within 30 min to over 2 h away.

### 2.5. Statistical Analysis

To assess each of health literacy and vaccine literacy on the respective nine-item scales, the average of the four-point score was used. The hours spent in care was taken to be the mid-point of each of the six categorical ranges up to 35 h, and 35 h for those reporting above 35 h, and thus treated as a continuous variable. The continuous variables were: age; household income; and hours of care provided to parents. The categorical variables were: gender; marital status; children; education; whether full-time or on care leave; parental care needs; and distance from parents. First, the variation of the difference of vaccine and health literacy with age was assessed, as age is a key risk factor for COVID-19. To analyze the variation with age, three subgroups were constructed based on age and the difference in vaccine and health literacy: under the age of 50; over the age of 50 and with vaccine literacy higher than health literacy; and over the age of 50 and with vaccine literacy lower than health literacy. Next, the two subgroups of those over 50 were compared to understand for which variables are the subgroups distinctive: this was to identify what variables are associated with higher difference in vaccine versus health literacy. The variables across the two subgroups were compared using Student’s *t*-test for continuous variables and chi-square test for categorical variables. This single-variable analysis was, finally, complemented by single-variable and multivariable regressions, assuming a normal distribution. The dependent variable was the difference in vaccine versus health literacy and the explanatory variables were the demographic and care-related variables. Turning to the media and information sources, the comparison of use of each media source to inform vaccine literacy versus health literacy was, as the variables are continuous, based on Student’s *t*-test. A *p*-value less than 0.05 was considered to indicate statistical significance for all analyses. The data were analyzed using STATA (Statistics Data Analysis, Version MP-13.1 for Windows, StataCorp LP, Texas, TX, USA).

## 3. Results

The sample of family carers for older parents who also work or are taking leave from work due to provision of care (*n* = 292) comprises 44% women and 56% men, with mean age 53 years (9.1 standard deviation). Participants’ living arrangements with respect to their parents receiving care vary, including 55% living with or close by their care-receiving parents. Also, their care-receiving parents span the range of the official seven care needs categories, from needing light daily-living support to requiring full-time assistance [29]. Participants’ self-reported hours spent on care average 8.3 h per week (9.7 standard deviation), with 44% under 3 h, 36% between 3–15 h, and 20% over 35 h.

**Figure 1:**
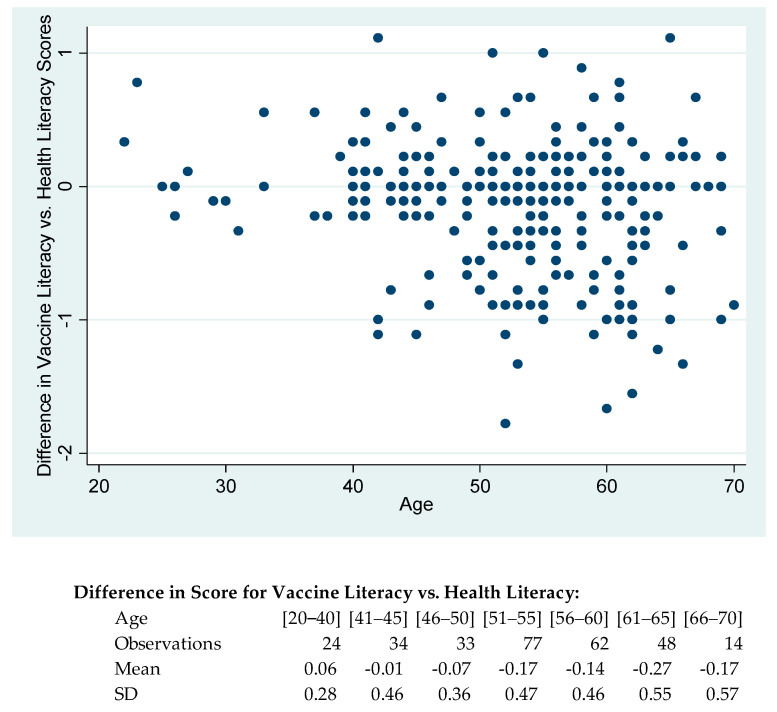
Difference in score for vaccine literacy versus health literacy with age of family carer.

Among participants, the mean score for vaccine literacy is slightly lower than for health literacy (mean of 2.73 versus 2.86, *p* = 0.004), with a 0.64 correlation. The mean scores across the nine items ranges for vaccine literacy from 2.55 to 2.84 and for health literacy from 2.69 to 2.94. The analysis compares individuals’ vaccine literacy versus their health literacy, as health literacy was asked about in general whereas vaccine literacy with respect to current COVID-19 vaccinations. Comparing the difference in score for vaccine and health literacy with participant characteristics reveals that as the age of participants increases, the difference between literacy scores decreases, with younger participants having on average higher vaccine literacy than health literacy whereas the opposite is the case for older participants; and the standard deviation increases, thus there is a greater variation across older participants (Figure 1). This is a striking pattern, given that the risk of COVID-19 rises sharply with age.

To understand the increased variability with age in the difference in vaccine and health literacy scores, first, three groups are compared (Table 1) in terms of their demographic and care characteristics: one group for those under the age of 50; and two groups for over the age of 50, one with higher and the other lower vaccine literacy versus health literacy score. Focusing on the two groups over the age of 50, the difference in the means of vaccine literacy is 0.7 (*p* < 0.001) and not significant for health literacy. Considering the other variables, the difference between the two groups is almost significant for having children (*p* = 0.066) and significant for hours of care provided to parents (*p* = 0.018).

These patterns of significance were analyzed through multivariable analysis with, as a dependent variable, the difference in vaccine and health literacy scores, and as independent variables the demographic and care-related variables (in Table 1): age; gender; marital status; whether or not have children; household income; educational level; whether full-time employees or taking care leave; parental care needs; and distance from parent; and hours of care provided to parent. The significant variables are (with coefficient and 95% confidence interval in square brackets, and with results available in the supplementary materials): age (−0.01 [−0.02 to −0.002]), children (0.14 [0.01 to 0.28]) and hours of care (0.01 [0.004 to 0.02]). As a comparison, for these variables the crude coefficients and 95% confidence intervals are: age (−0.01 [−0.01 to −0.003]), children (0.16 [−0.09 to 0.13]) and hours of care (0.01 [0.01 to 0.02]). Thus, at a given age, higher vaccine literacy relative to health literacy is associated with having children (in multivariable analysis) and increased hours of care.

Turning to analyze the sources of information used to inform vaccine literacy (Table 2), the average number of sources used is 3.3 compared to 3.5 for health literacy, with small difference −0.2 nonetheless significant (*p* = 0.03). There are, however, significant differences in the mix of sources used. For vaccine literacy the main sources that more than 20% of participants are using are (with multiple responses possible): national television (56%), social media (37%), doctors (35%), national newspapers (29%), local television (27%), family (24%), and local newspapers (20%). In contrast, for health literacy the main sources are: social media (47%), national television (46%), doctors (46%), family (30%), national newspapers (29%), care managers (27%), friends (27%), and local newspapers (21%). Overall, participants use for vaccine literacy, compared to health literacy, more mass media and less inter-personal sources, with the proportion of participants indicating as a source: 10% higher for national television (*p* < 0.001), 6% higher for local television (*p* = 0.01), 4% higher for local newspapers (*p* = 0.03), 9% lower for social media (*p* < 0.001), 11% lower for doctors (*p* < 0.001), 9% lower for care-managers (*p* < 0.001), 7% lower for friends (*p* < 0.001) and 5% lower for family (*p* = 0.03). Overall, the mix of sources for vaccine literacy versus health literacy is rated to be similarly useful (3.2) and slightly less reliable (3.2 for vaccine literacy versus 3.3 for health literacy, with a difference of −0.1 with *p* = 0.01).

Considering the three different groups (participants under the age of 50; over the age of 50 with higher or lower difference in vaccine literacy versus health literacy), there are no significant differences in sources of information used. Thus, for those over the age of 50 the significant differences in vaccine literacy relative to health literacy are not explained by access to different sources of information. Also, the assessment of overall reliability and usefulness of these sources does not differ across these groups. Hence, the difference in vaccine literacy could be from how participants encode such information and how such information affects their actual actions. Considering the nine items to assess vaccine literacy provides an indication. For those over the age of 50 comparing higher versus lower vaccine literacy, while all nine items have statistically significant differences in average value, the two items with the largest magnitude difference in vaccine literacy relate to whether the participant will take action. One item asks whether participants have taken or will take action following the knowledge and information they received (e.g., book a vaccination when an appointment becomes available) and the other relates to whether through their information search they decide by themselves if they want to be vaccinated. Thus, the main difference between these groups would stem from their ability to decide to act based on the information accessed.

## 4. Discussion

The survey participants are drawn from family carers who work and provide care for their older parents. Thus, they are both in close contact with their parents as well as at the intersection of several social domains, including other family members, colleagues at work and community. Given that the risk from COVID-19 increases with age and social contacts are important in transmission of the virus, such a set of participants is fundamental to understand for COVID-19 vaccination. The results are that as participants’ age increases, however, vaccine literacy relative to their own health literacy decreases and, importantly, is more variable. By comparison, in surveys covering a broader population, not only family carers, the impact of age on willingness to receive a COVID-19 vaccination has been mixed, both positive and negative [30,31], although neither study discussed whether variation in willingness to receive vaccination increases with age.

For family carers, the results are that as age increases there is a larger proportion of participants with vaccine literacy lower than their health literacy. Nonetheless, the risk of COVID-19 increases sharply with age, which would suggest the importance of higher vaccine literacy among older participants. As carers risk transmitting COVID-19 to the parents they care for and may be expected to influence their parents’ vaccine uptake, the relative gaps in vaccine literacy of some participants are of concern for potentially significant knock-on effects. At the same time, in response to the ageing of society, the approach in Japan to achieving resilient and sustainable social systems places emphasis on the continued involvement of such family carers. Interestingly, the findings are that those who spend more time caring for their parents, and those who have children, tend to have higher vaccine literacy. In contrast, neither physical proximity to their parents, including living together, nor the extent of care needed by their parents are associated with higher vaccine literacy. Rather, it is the extent of personal engagement in care that is associated with greater vaccine literacy. Evidence from a different broader sample survey across Japan points to the willingness to protect others as a motivation for intention to take-up COVID-19 vaccines [17]. As for family carers the results are that vaccine literacy is higher with more intense involvement in care for their parents, this suggests that motivations to protect others necessitate close involvement. If this were more generally the case, this would indicate potential limitations to appeals for vaccination take-up based on benefits to the broader community.

The information used to inform COVID-19 vaccine literacy is recognized as an urgent, critical aspect [32] and should recognize, as for vaccines in general, the plurality of sources used even though these differ in trust and reliability [26]. Indeed, at this stage in the pandemic and the start of vaccination roll-out in Japan, participants’ use of information sources is markedly different to understand COVID-19 vaccines as compared to health literacy in general. Consequently, communication strategies to address vaccine literacy should differ from those for health literacy, including whom to target and through which channels. The results indicate participants relying for vaccine literacy more on mass media, including national television, local television and newspapers, and less on social media and inter-personal sources of information, including family, colleagues, and health and care professionals. Clearly, the mix of information sources accessed may shift as the pandemic progresses and the vaccination roll-out gains pace in Japan.

The current increased use of mass media likely reflects the novel, fast moving dynamic of the virus and the desire to access credible sources [33]: furthermore, the increase in local newspapers and local television points to a desire for context-specific information. Also, such mass media sources preclude dialogues to support understanding and hence issues of information comprehension and absorption come to the fore, as has been documented in the U.S. for COVID-19 tests [34]. While the mix of media and information sources influences perspectives [25], there is also the opportunity for the mix of sources to be proactively adapted. Indeed, one interpretation for the different mix of sources used by participants for COVID-19 vaccine literacy, relative to their usual sources for health literacy, is as a means to achieve the similar overall degree of usefulness and reliability that they report.

At present, relative to health literacy, there is less access to person-specific information, which may become more important as vaccine roll-out progresses. The distinct pattern of media sources, relative to health literacy, points to the importance of an integrated media approach to support community-wide vaccine campaigns. Furthermore, among family carers over the age of 50, those with higher vaccine literacy had particularly higher self-rating on items related to the propensity to act on the information received: hence, further research could address how campaigns to improve vaccine literacy could enable and support the transition to act on the information received (e.g., to book a vaccination appointment, to attend the appointment, to complete the vaccination) complemented by understanding of how to make the corresponding processes and systems (e.g., how to book a vaccination appointment) supportive of those with lower vaccine literacy.

The online survey format necessarily has some limitations, such as participants needing to be registered with the survey company and internet users thus limiting external validity, although it does enable timely insights. Furthermore, there is an ability to address specific groups of interest, such as the family carers focused on that is an important group for vaccine roll-out due to their role in caring for their older parents. As all the respondents are Japanese (which reflects that 98% of the population in Japan is Japanese [35]), there is no significant scope for race or cross-cultural subsamples to consider. More generally, the survey points to the value of understanding the specific dynamics related to vaccine understanding and take-up for distinct segments of the population.

Indeed, vaccine literacy is evidenced to have important marks of distinction relative to health literacy, including which personal and social characteristics impact vaccine literacy and what sources of information are most distinctively accessed. These findings support the call for further understanding on vaccine literacy solely and in combination with health literacy, not least during the current pandemic and, more generally, contribute towards achieving a sustainable and resilient society.

## Figures and Tables

**Table 1:** Demographic, work and care characteristics for sub-samples based on age and difference in vaccine literacy versus health literacy.

	**Whole Sample**			**Age Over Age 50 and Vaccine Literacy Higher than Health Literacy**		
Variable	Observations (*n*)	Mean	Std. Dev.	Observations (*n*)	Mean	Std. Dev.
Vaccine Literacy (1 to 4)	292	2.73	0.60	107	3.04	0.39
Health Literacy (1 to 4)	292	2.86	0.48	107	2.87	0.42
Difference between Vaccine Literacy versus Health Literacy	292	−0.13	0.47	107	0.17	0.25
Age	292	52.9	9.12	107	57.8	4.84
Gender (Woman = 1; Man = 0)	292	0.44	0.50	107	0.49	0.50
Marital status (Married = 1; Other = 0)	292	0.67	0.47	107	0.73	0.45
Children (Yes = 1; No = 0)	292	0.62	0.49	107	0.75	0.44
Household Income (10,000 Yen per year)	292	720	443	107	752	421
Education level (% college or higher education)	292	0.26	0.44	107	0.22	0.42
Employment (% full-time not taking care leave)	292	0.88	0.33	107	0.91	0.29
Parents care needs (% with lighter need: support at levels 1 or 2, or care at level 1 of 5, on government scale)	292	0.57	0.50	107	0.57	0.50
Distance from home to parents home: % living together or within walking distance	292	0.55	0.50	107	0.58	0.50
Care provided to parent (hours per week)	292	8.31	9.70	107	9.45	10.29
	**Age under age 50**			**Age over age 50 and vaccine literacy lower than health literacy**		
Variable	Observations (*n*)	Mean	Std. Dev.	Observations (*n*)	Mean	Std. Dev.
Vaccine Literacy (1 to 4)	91	2.73	0.57	94	2.37	0.62
Health Literacy (1 to 4)	91	2.74	0.53	94	2.95	0.48
Difference between Vaccine Literacy versus Health Literacy	91	−0.01	0.38	94	−0.58	0.38
Age	91	42.3	7.12	94	57.7	4.74
Gender (Woman = 1; Man = 0)	91	0.41	0.49	94	0.43	0.50
Marital status (Married = 1; Other = 0)	91	0.54	0.50	94	0.72	0.45
Children (Yes = 1; No = 0)	91	0.46	0.50	94	0.63	0.49
Household Income (10,000 Yen per year)	91	703	491	94	698	421
Education level (% college or higher education)	91	0.22	0.42	94	0.35	0.48
Employment (% full-time not taking care leave)	91	0.90	0.30	94	0.83	0.38
Parents care needs (% with lighter need: support at levels 1 or 2, or care at level 1 of 5, on government scale)	91	0.60	0.49	94	0.52	0.50
Distance from home to parents home: % living together or within walking distance	91	0.47	0.50	94	0.59	0.50
Care provided to parent (hours per week)	91	9.01	10.47	94	6.35	7.86

**Table 2:** Sources of information used to inform vaccine and health literacy.

**(a) Proportion of respondents using the source of information**					
	**Vaccine Literacy**		**Health Literacy**		**Difference in Means: Vaccine-Health Literacy**
Sources of information	Mean	Std. Dev.	Mean	Std. Dev.	
Newspapers: National editions	29%	45%	29%	46%	−0.3%
Newspapers: Local editions	20%	40%	15%	36%	4.5%
TV: National channels	56%	50%	46%	50%	9.9%
TV: Local channels	27%	45%	21%	41%	5.8%
Radio: National channels	9%	28%	8%	26%	1.0%
Radio: Local channels	9%	29%	8%	28%	0.7%
Social Media	37%	48%	47%	50%	−9.2%
Podcasts	4%	21%	4%	19%	0.7%
Colleagues	16%	37%	17%	37%	−0.7%
Bosses and companies	13%	34%	14%	35%	−1.0%
Doctors	35%	48%	46%	50%	−11.0%
Care manager or care worker	17%	38%	27%	44%	−9.2%
Friends	21%	40%	27%	45%	−6.5%
Family	24%	43%	30%	46%	−5.5%
Others	5%	23%	8%	27%	−2.4%
Average number of sources used	3.3	2.7	3.5	2.7	−0.2
**(b) Respondents rating of reliability and usefulness of source of information used (from 1 = not at all to 5 = very much)**					
Reliability	3.23	0.89	3.33	0.87	−0.11
Usefulness	3.21	0.89	3.28	0.85	−0.07

Note: 292 observations for each item in (a) and (b).

## Data Availability

The data presented in this study are openly available in SciencesPo registry upon publication and up to then upon request from author.

## Supplementary Materials

The following are available online at www.mdpi.com/article/10.3390/healthcare9081038/s1, Table S1: Regression results from multivariable regression, Table S2: Regression results from single-variable regressions.
