# Supplementary material for: COVID-19 Vaccine Literacy of Family Carers for Their Older Parents in Japan
Source: Healthcare (Basel). 2021 Aug 12;9(8):1038. doi: 10.3390/healthcare9081038 (PMC8393727; doi:10.3390/healthcare9081038)
Supplement: Supplementary file 1 [file healthcare-09-01038-s001.zip › healthcare-1280059-supplementary.pdf]

Supplementary Materials

Supplementary Table S1. Regression results from multivariable regression.

**Dependent variable: Difference between Vaccine Literacy and Health Literacy**				
	**Coef.**	***p*-value**	**[95% Conf. Interval]**	
Age	−0.01	0.01	−0.02	0.00
Gender (Woman = 1; Man = 0)	0.03	0.60	−0.09	0.15
Marital status (Married = 1; Other = 0)	−0.04	0.61	−0.18	0.11
Children (Yes = 1; No = 0)	0.14	0.04	0.01	0.28
Household Income (Yen 10,000 per year)	0.00	0.37	0.00	0.00
Education level (% college or higher education)				
2	0.20	0.32	−0.19	0.59
3	0.31	0.16	−0.12	0.74
4	0.18	0.39	−0.23	0.58
5	0.31	0.11	−0.08	0.70
6	0.09	0.70	−0.36	0.54
7	0.41	0.29	−0.35	1.16
Employment (% full-time not taking care leave)				
2	−0.09	0.34	−0.27	0.09
3	−0.18	0.44	−0.65	0.28
Parents care needs (% with lighter need: support at levels 1 or 2, or care at level 1 on 5-point scale)				
2	0.11	0.12	−0.03	0.26
3	0.00	0.99	−0.15	0.15
4	−0.02	0.85	−0.22	0.18
5	−0.13	0.25	−0.35	0.09
6	0.04	0.73	−0.20	0.29
Distance from home to parents home: % living together or within walking distance				
2	−0.11	0.22	−0.28	0.06
3	0.02	0.77	−0.13	0.17
4	−0.07	0.42	−0.26	0.11
5	−0.04	0.73	−0.29	0.20
6	0.08	0.45	−0.13	0.30
Care provided to parent (hours per week)	0.01	0.00	0.00	0.02
Constant	−0.01	0.97	−0.51	0.49

Supplementary Table S2. Regression results from single-variable regressions.

Dependent variable: Difference between Vaccine Literacy and Health Literacy				
	**Crude Coef.**	***p*-value**	**[95% Conf. Interval]**	
Age	−0.01	0.00	−0.01	0.00
Gender (Woman = 1; Man = 0)	0.03	0.60	−0.08	0.14
Marital status (Married = 1; Other = 0)	−0.09	0.13	−0.20	0.03
Children (Yes = 1; No = 0)	0.16	0.77	−0.09	0.13
Household Income (Yen 10,000 per year)	0.00	0.85	0.00	0.00
Education level (% college or higher education)				
2	0.26	0.19	−0.13	0.65
3	0.37	0.08	−0.05	0.79
4	0.29	0.15	−0.11	0.69
5	0.41	0.04	0.03	0.79
6	0.20	0.37	−0.24	0.65
7	0.46	0.22	−0.28	1.21
Employment (% full-time not taking care leave)				
2	−0.09	0.34	−0.26	0.09
3	−0.10	0.66	−0.57	0.36
Parents care needs (% with lighter need: support at levels 1 or 2, or care at level 1 on 5-point scale)				
2	0.15	0.05	0.00	0.29
3	0.05	0.53	−0.10	0.20
4	0.02	0.82	−0.17	0.22
5	−0.06	0.57	−0.28	0.15
6	0.15	0.23	−0.10	0.40
Distance from home to parents home: % living together or within walking distance				
2	−0.10	0.09	−0.27	0.07
3	0.01	0.07	−0.13	0.16
4	−0.07	0.09	−0.25	0.10
5	−0.04	0.12	−0.29	0.20
6	0.02	0.11	−0.19	0.24
Care provided to parent (hours per week)	0.01	0.00	0.01	0.02
